# The developmental basis of adult arrhythmia: atrial fibrillation as a paradigm

**DOI:** 10.3389/fphys.2013.00221

**Published:** 2013-09-12

**Authors:** Sunil Kapur, Calum A. MacRae

**Affiliations:** Medicine, Cardiovascular Division, Brigham and Women's Hospital and Harvard Medical SchoolBoston, MA, USA

**Keywords:** arrhythmia, developmental biology, genetics, electrophysiology, remodeling

## Abstract

Normal cardiac rhythm is one of the most fundamental physiologic phenomena, emerging early in the establishment of the vertebrate body plan. The developmental pathways underlying the patterning and maintenance of stable cardiac electrophysiology must be extremely robust, but are only now beginning to be unraveled. The step-wise emergence of automaticity, AV delay and sequential conduction are each tightly regulated and perturbations of these patterning events is now known to play an integral role in pediatric and adult cardiac arrhythmias. Electrophysiologic patterning within individual cardiac chambers is subject to exquisite control and is influenced by early physiology superimposed on the underlying gene networks that regulate cardiogenesis. As additional cell populations migrate to the developing heart these too bring further complexity to the organ, as it adapts to the dynamic requirements of a growing organism. A comprehensive understanding of the developmental basis of normal rhythm will inform not only the mechanisms of inherited arrhythmias, but also the differential regional propensities of the adult heart to acquired arrhythmias. In this review we use atrial fibrillation as a generalizable example where the various factors are perhaps best understood.

## Introduction

Atrial fibrillation (AF) is the most common cardiac arrhythmia (Go et al., 2001) and is associated with substantial morbidity and mortality even in paroxysmal forms (Benjamin et al., 1998). AF is a central phenotype at the nexus of a broad spectrum of cardiovascular syndromes including; hypertension, congestive heart failure (CHF), and thromboembolism. AF is typically associated with different forms of structural heart disease, ischemic or other cardiomyopathies and systemic disorders, so that the mechanism of the arrhythmia has been variously attributed to elevated atrial pressures, chronic atrial fibrosis or inflammatory stimuli (Nattel et al., 2000). However, not unlike many other common clinical arrhythmic phenotypes, it has proven remarkably difficult to discriminate primary from secondary phenomena in the causal chain. In the last few years a confluence of basic and translational research has begun to define a role for a primary underlying diathesis in many, if not all, forms of AF (Ellinor et al., 2008; Postma et al., 2009). In this review we will summarize recent data in this area, highlighting developmental aspects of the biology of AF and the relationship to downstream biology including ventricular dysfunction that predisposes to other arrhythmias and to sudden death. We will focus on the importance of exploring the basis of arrhythmias using approaches that not only encompass all of the native biological context, but that also allow the characterization of the very earliest and most upstream pathophysiologic events.

## A primary diathesis

While the mechanism of sustained AF is unclear, many potential factors have been proposed. Three models have been considered for nearly 100 years (Garrey, 1924), though the precise relationship of each of these conceptual frameworks to human AF is still an area of investigation. The focal mechanism theory with fibrillatory conduction stands on the notion that AF is provoked by the rapid firing of single or multiple ectopic foci (Waldo, 2003), and also suggests a role for continued ectopic firing in the maintenance of AF. This theory has gained recent support from clinical observations (Haissaguerre et al., 1998). The single circuit re-entry theory of AF assumes the presence of a single dominant reentry circuit—a “mother rotor” with the fragmentation of emanating waves in the heterogeneous electrical substrate of normal atrial tissue. The multiple wavelet theory of AF assumes the presence of multiple reentry circuits with randomly propagating wavefronts that must find receptive tissue in order to persist. Shortening of the refractory period of atrial myocytes and slowing of conduction velocities—central features of the electrical remodeling seen with AF—both may help to stabilize the arrhythmia by decreasing circuit size (Ausma et al., 2001). Clearly these three mechanistic models are not mutually exclusive and each may be applicable to certain subgroups of AF patients or may coexist in a single subject at various stages in the pathogenesis of AF (Patton et al., 2005).

There are numerous animal models of AF, each of which capture some aspects of the underlying biology (Milan and MacRae, 2005). However, most AF models, if not all, require some continued extrinsic stimulus for the maintenance of the arrhythmia. The extrinsic stimuli may include vagal stimulation, continuous atrial pacing, or combinations of different manipulations. Irrespective of the stimulus, on withdrawal there is almost always reversion to normal sinus rhythm, usually within hours. Together these observations suggest that the spontaneous paroxysmal or persistent AF observed in susceptible humans is the result of a fundamental predisposition to the arrhythmia upon which acquired or environmental factors are layered. While a large number of experimental studies have explored the mechanisms of both the onset and maintenance of the arrhythmia over the last few decades, there remains little understanding of the biologic basis of this presumed underlying primary diathesis. A fundamental premise of this review is that each of the known mechanisms operant in later stages of AF may be influenced by developmental pathways, such that this apparently late onset arrhythmia may be conditioned by intrinsic differences in the excitability of the pulmonic veins, the coupling patterns of atrial myocytes, the electrophysiologic properties of individual cardiomyocytes or any other contributory factor determined during the earliest stages of cardiogenesis.

Further evidence in support of a primary predisposition to AF has emerged from studies of the genetics of different forms of the arrhythmia. Traditionally, AF has not been considered a genetic condition. However, a number of recent studies have demonstrated that some forms of the arrhythmia, and in particular lone AF, have a substantial genetic basis (Ellinor et al., 2005, 2008; Sinner et al., 2011). These findings raise the possibility that, in at least a subset of AF, susceptibility to the arrhythmia is a function of intrinsic biologic differences in the hearts of affected individuals.

Together, these data supporting an intrinsic predisposition toward AF infer that our understanding of the arrhythmia is likely to benefit from exploration of the developmental biology of atrial electrophysiology. This approach is particularly powerful in the context where the known effects of the arrhythmia itself on atrial physiology and cell biology-so called atrial remodeling-obscure the distinction between cause and effect. The opportunity to define the earliest events in AF, before these have been contaminated by environmental modifiers or the remodeling associated with even short-lived episodes of the arrhythmia itself, offers the opportunity for unique insights into the potential biologic mechanisms of spontaneous or sustained arrhythmia, as well as the complex links between this “benign” atrial disorder and CHF or stroke.

## Modern clinical insights

Seminal clinical studies by Haissaguerre et al. (1998) demonstrated that in a subset of paroxysmal AF may be initiated by repetitive rapid electrical activity originating from the pulmonary veins (PVs), and that ablation of such foci might “eliminate” paroxysmal forms of the arrhythmia. Post-mortem anatomical studies showed that PV myocardial sleeves were present in 100% of patients with AF, whereas similar muscle tissue was observed in only 85% of those without AF (Hassink et al., 2003). In addition, patients with AF had significantly longer muscle sleeves with considerably thicker PV myocardial tissue. In some instances arrhythmogenicity could only be localized to these areas of thickening (Guerra et al., 2003). While in most cases the electrical origins of paroxysmal AF are found in the myocardial sleeves clothing the pulmonary veins (Nattel, 2003), additional sites for ectopic automaticity are increasingly recognized. For example, triggered and automatic activity can be induced in the muscle sleeves of the canine coronary sinus, but not in the atrial cells. Other investigators have noted that other venous sites, such as the superior vena cava and the vein of Marshall, can also serve as the origin of rapid ectopy driving AF. It is known that the thoracic veins activate at shorter activation cycle lengths than the atria during sustained AF in dogs (Wu et al., 2001). These findings suggest that rapid activity from thoracic veins might be responsible for the perpetuation of non-paroxysmal AF (Chen et al., 2002a). Compatible with this hypothesis, many clinical studies have shown that RF ablation or isolation of thoracic veins may result in the elimination at least for some time of chronic AF in humans. These human data suggest that AF may be the result of perturbation of pathways that normally suppress automaticity in veins connecting to the atria or prevent conduction of electrical impulses from the veins to the atria.

## Patterning the atrium and its boundaries

### The atria form from distinctive populations of cardiomyocytes

The four-chambered mammalian heart develops from a simple linear tube. Embryologic studies have shown that the complicated looping process of the heart tube brings together the essential parts of the sino-atrial and primary ring myocardium which are the embryonic precursors of definitive conduction system (van den Berg et al., 2009). The myocardium of the original heart tube is only part of the eventual heart, as further myocardium is subsequently added to its poles. A hallmark feature of the primary myocardium is the expression of connexins (Cx) 30.2 and 45, which form low-conductance gap-junction channels. The transcriptional repressor genes Tbx2 and Tbx3 maintain the phenotype of primary myocardium (Habets et al., 2002). In contrast, chamber myocardium, as it is specified, expresses Cx40 and Cx43, which form high-conductance gap-junction channels, and the α-subunit of the sodium channels Nav 1.5, encoded by *Scn5a*, providing the atrial compartment with the excitability necessary for high conduction velocities. During atrial septation the pulmonary orifice is positioned in the left atrium, and the smooth-walled dorsal atrial wall forms from cardiac precursor cells recruited from the pulmonary mesenchyme, which express the transcription factors Nkx2-5 and Isl1 (Mommersteeg et al., 2007a; Snarr et al., 2007). As organogenesis progresses the pulmonary venous component becomes incorporated into the roof of the left atrium, such that each of the four PVs, with short myocardial sleeves of various lengths, opens at a corner of the atrial back wall. This integration of venous and atrial cells appears to be the result of highly conserved guidance cues paralleling those in the central and peripheral nervous systems. From the outset, the so-called pulmonary myocardium expresses *Cx40* as observed in working myocardium, although it does not express *Nppa*, the gene encoding atrial natriuretic peptide. The myocardium that is added at the systemic part of the venous pole, or sinus venosus myocardium, is derived from Nkx2-5-negative, Isl1-negative, and Tbx18-positive mesenchymal cardiac precursors (Christoffels et al., 2006). This sinus venosus myocardium starts to form at day 9.5 of mouse development and initially is Cx40-negative, but expresses the pacemaker channel Hcn4 and the transcriptional repressor Tbx3 (Hoogaars et al., 2004). Ultimately, the sinus myocardium matures into atrial working myocardium, with the exception of the region where the sinus node is formed, which remains Tbx3-positive, Nkx2-5-negative, Cx40-negative, and Hcn4-positive. Notably, these data suggest that distinctive cardiomyocyte populations with discrete automaticity and excitability, as well as coupling characteristics are determined at the boundaries of the cardiac chambers under the regulation of combinatorial patterns of transcription factor expression. Thus, several regions, including the orifice of the coronary sinus, the terminal crest, and the lower rim of the right atrium, are functionally distinct from the remainder of the atrium and represent the core physiologic and anatomical substrate for specific atrial arrhythmias. These same myocardial regions are often involved in structural congenital heart disease, explaining to some extent the frequent occurrence of atrial flutter and AF in these disorders. Whether this heterogeneity extends into the chamber myocardium remains to be seen, but the very same signaling pathways are deployed in the complex patterning of neurogenesis suggesting this is likely.

### Other cell types contribute to atrial physiology

Embryological evidence also supports the participation of other cell types in genesis of normal atria. Among these are cells with features of specialized conduction system within PV myocardium. Investigators have noted the presence of spontaneously depolarizing cells in the PVs of dogs (Chen et al., 2000). Recently, “P cells,” normally found in the AV and SA node, have also been localized to the PVs (Perez-Lugones et al., 2003; Wit and Boyden, 2007). These were found in substantially greater numbers near the atrial ostia. The same study also identified Purkinje and transitional cells in PVs. The current belief is that spontaneous depolarization in P cells may lead to the production of electrical impulses that are propagated to the left atrium through Purkinje-like cells (Chen and Yeh, 2003).

Studies have also demonstrated that cells with gene expression features consistent with developing cardiac conduction system could be found in PVs prior to birth (Jongbloed et al., 2004). In a CCS-lacZ transgenic line, LacZ positive cardiomyocytes were observed in the PVs (Rentschler et al., 2001). The heterogeneous presence of CCS-lacZ expression was also noted in other tissue involved in atrial arrhythmias, including Bachmann's bundle (Perez-Lugones et al., 2003).

The situation is further complicated by the identification of cells resembling the so-called interstitial cells of Cajal in the muscular pulmonary venous sleeves from at least some human subjects (Morel et al., 2008). Although their exact function is unclear, correlation with phenotypically similar cells in the gut, as well as direct observations, leave little doubt that these cells are capable of providing a pacemaking function (Thuneberg, 1982; Sanders, 1996). Unlike the situation in the heart, or in the renal tract (Gosling and Dixon, 1972), the interstitial cells within the gut are not grouped together into “nodes,” but rather intermingle with the myocytes in the intestinal wall. Similarly, the interstitial cells in the PVs appear to comingle with the myocytes making up venous sleeves. Their potential pacemaking function is demonstrated by their reactivity with HCN4, a marker for the hyperpolarization-activated and cyclic nucleotide-sensitive ionic current.

Recent seminal work has defined pigmented cells within the murine heart, though their function remains unknown (Mjaatvedt et al., 2005). Interestingly, dermal melanocytes and melanoma lines do express voltage-dependent currents and under conditions of increased oxidative stress some ionic currents are modified to promote cellular excitability (Levin et al., 2009). The melanin synthesis enzyme dopachrome tautomerase (Dct) contributes to, but is not required for, melanin synthesis in dermal melanocytes by catalyzing the conversion of L-dopachrome to 5,6-dihydroxyindole-2-carboxylic acid which is thought be an intermediate in the eumelanin synthesis pathway. Furthermore, Dct is important for intracellular calcium regulation; in fact, eumelanin binds calcium with an affinity similar to calmodulin (Bush and Simon, 2007). Studies have shown a unique Dct-expressing cell population within murine and human hearts is specifically located around the pulmonary veins, atria, and atrioventricular canal. Adult mice lacking Dct displayed normal cardiac development but an increased susceptibility to atrial arrhythmias. Cultured primary cardiac melanocyte-like cells were excitable, and those lacking Dct displayed prolonged repolarization with early afterdepolarizations. Furthermore, mice with mutations in the tyrosine kinase receptor Kit lacked cardiac melanocyte-like cells and did not develop atrial arrhythmias in the absence of Dct. These data suggest that dysfunction of melanocyte-like cells in the atrium and pulmonary veins may contribute to atrial arrhythmias (Levin et al., 2009).

Neuronal contributions to the final developmental cellular biology of the atria are complex. The intrinsic nervous system of the heart is incompletely characterized and its role in AF remains largely speculative (Huang et al., 1996). However, there is no doubt that these cells and the associated autonomic ganglia play a central role in the control of atrial excitability later in life (Verrier and Antzelevitch, 2004). The emerging links between autonomic innervation and AF with therapeutic manipulation through radiofrequency ablation, both of cardiac and renal plexuses, suggest that there is much still to be learned (Pokushalov et al., 2012).

## Specification of cellular function

Foci of abnormal automaticity are the result of either (1) an abnormal pacemaker discharging spontaneously in the absence of an initiating stimulation or (2) automatic activity triggered by an initiating stimulus related to oscillating after-depolarization or early after-depolarization. The venous-atrial boundary is also a potential nidus for abnormal electrical activity (Postma et al., 2009). Structurally, the transition from atrial to venous walls is gradual as the left atrial myocardial sleeves overlap with smooth muscle of the venous myocardium (Ho et al., 1999). The muscle fibers in pulmonary veins are usually orientated perpendicularly to the blood flow. Such an arrangement, together with increased anisotropy due to ageing-induced fibrosis, may facilitate re-entry within the pulmonary veins. Further, the cellular properties of PV cardiomyocytes, such as shorter action potential and lower phase-0 upstroke velocities than in the left atrium, favor re-entry (Ehrlich et al., 2003). Quantitative mapping analysis of the left atrial-PV ostium junction indicates impulse conduction block or delay at sites noted for the presence of connective tissue barriers. The fact that connective tissue septae separating myocyte tissue islands are much more common in the PVs as compared with the atria may contribute to the role of PV ectopy in arrhythmogenesis (Hamabe et al., 2003).

Beyond the ectopic development of pacemaker cells, perturbation of the electrophysiologic specification of PV cardiomyocytes may occur leading to intrinsic abnormalities of automaticity. Normal PV myocytes may display spontaneous phase 4 depolarizations, along with slowed phase 0 rates, both characteristic features of pacemaker cells in the SA node (Chen et al., 2000). The PV myocyte membranes have a much lower density of I_*K*1_-channels as compared with typical atrial tissue (Chen et al., 2001). SA nodal cells also have a characteristic I*_f_*-current, whose presence was recently demonstrated within PV myocytes (Chen et al., 2001), allowing for pacemaker behavior in PV cells. Another possibly important finding was the presence of I_Ca2+−t_-channels within PV myocytes (Chen et al., 2004). The Ca^2+^-current from these channels has been shown to aid in the production of spontaneous depolarizations by causing the release of Ca^2+^ from the sarcoplasmic reticulum at low voltages (−60 mV), facilitating pacemaker activity. These channels may also increase the plateau phase of action potentials, raising the possibility of EADs. Application of nickel, a rather non-specific inhibitor of I_Ca2+−t_-channels, to PV tissue drastically reduced both triggered activity and automaticity in PV myocytes (Huser et al., 2000).

As noted, there is quite extensive innervation of the PVs and atria (Gardner and O'Rahilly, 1976). Functionally, heart rate variability analyses note that sympathovagal imbalance is present before the onset of paroxysmal AF episodes. The importance of autonomic innervation is further supported by animal experiments and recent clinical studies showing that vagal denervation enhances the efficacy of circumferential pulmonary vein ablation in preventing AF recurrence (Pokushalov et al., 2012). ANS activation facilitates early afterdepolarization and triggered activity by simultaneously prolonging the intracellular calcium [Ca(i)] transient (sympathetic effect) and shortening the action potential duration (parasympathetic effect) (Chen and Tan, 2007). Beyond the immediate functional implications, it is possible that autonomic innervation has trophic effects on cardiac development (Postma et al., 2009).

### Patterning of cellular connections

An interesting potential connection between left-right asymmetry and AF has recently emerged though studies of developmental biology and the human genetics of AF. Left-right asymmetric morphogenesis is initiated in the presomitic embryo and is mediated through the Nodal signaling pathway a major downstream effector of which is the Pitx2 homeobox gene (Hamada et al., 2002). One isoform of this protein, Pitx2c, is required for left–right patterning of the visceral organs, including the atria and systemic venous return of the heart (Franco and Campione, 2003). Human genetic studies have identified sequence variants on chromosome 4q25, close to the *Pitx2* gene that are strongly associated with increased risk for AF, making this a likely candidate for the predisposition to AF at this locus (Gudbjartsson et al., 2007). PITX2 is known to act in cardiac development by directing asymmetric morphogenesis of the heart (Gudbjartsson et al., 2007). Knockout models of PITX2 in mice have been shown to de-repress the normal inhibition of sinoatrial node formation in the left atrium (Mommersteeg et al., 2007b). PITX2 was also demonstrated to be necessary for the development of the pulmonary myocardium (Mommersteeg et al., 2007a). Pitx2c-deficient mice do not develop a pulmonary myocardial sleeve because they fail to form the initial pulmonary myocardial cells (Mommersteeg et al., 2007a).

From the outset, pulmonary myocardium expresses the transcription factor Nkx2-5 and its target gap-junction gene Cx40. Notably, when Nkx2-5 protein levels were lowered experimentally, the pulmonary myocardium transitioned to a Cx40-negative, Hcn4-positive phenotype, resembling sinoatrial nodal-like cells. Thus, under a reduced dose of a single transcription factor, Nkx2-5, the pulmonary myocardium converts more easily into a nodal phenotype, suggesting that subtle genetic variation between individuals in Nkx2-5 dosage could be an important contributing trigger to the development of AF (Postma et al., 2009). Indeed, some mutations in Nkx2-5 are suggested to be linked to AF (Gutierrez-Roelens et al., 2006), though much as with the Pitx2 story, the formal causal connection with human disease is not complete. Taken together, variations in the regulatory sequences of transcription factors specifying the final physiologic characteristics of cardiomyocytes throughout the entire cardiac anlagen are prime candidates for further research into the underlying cause of AF.

Other developmental pathways have also been implicated in the patterning of cardiomyocyte connections including the Wnt family of developmental signaling molecules. These secreted proteins regulate crucial aspects of development, including cell-fate specification, proliferation, survival, migration, and adhesion (Nusse, 2005). The effects of canonical Wnt/β-catenin signaling on cardiac neural crest cells may be mediated by both the transcriptional upregulation and functional activation of *Pitx2* (Malekar et al., 2010). Again the picture that is emerging is one of complex and coordinated specification of cellular physiology and cellular connectivity, with the potential for additional functional remodeling during development and beyond. Extensive systems-level studies will be necessary to define the gene networks and the functional inputs responsible for the final atrial phenotypes in health and disease (Brundel et al., 2002).

## Perpetuation of AF: intrinsic differences in PV and atrial substrate

While an active focus may be needed to initiate AF, re-entrant circuits are needed to maintain it. Persistent AF is characterized by the presence of re-entry pathways. Shorter refractory periods facilitate the maintenance of AF by increasing the prospect for re-entry. Cardiomyocytes of PVs exhibit varying refractory periods that may lend themselves well to the development of AF (Jais et al., 2002; Tada et al., 2002), but the electrophysiologic basis of these altered refractory periods in the PV is not fully accounted for. PV cell membranes exhibit an increased I*_Ks_*- and I*_Kr_*-current densities, as well as reduced I_Ca2+_-currents, when compared to neighboring left atrial myocytes (Ehrlich et al., 2003). In addition, the cognate channel proteins were present at greater concentrations within PV myocytes (Melnyk et al., 2005). These observations suggest that individuals with inherited variations in PV myocyte channel protein expression or function may be predisposed to AF.

There is robust genetic evidence that primary gain-of function mutations in ion channels can mediate increased I*_Ks_*, resulting in familial AF (Chen et al., 2003a). From a four-generation family with AF, the investigators were able to map the disease locus to a 12-megabase region on the short arm of chromosome 11. The KCNQ1 gene was located within this region and sequencing of the gene revealed a serine to glycine missense mutation at position 140 (S140G) in affected family members. The S140G mutation is located in the first transmembrane-spanning segment at the outer edges of the voltage-sensing domain and far from the pore-forming region of the potassium channel structure (Schenzer et al., 2005). Unlike the mutations in KCNQ1 associated with the long QT syndrome, which typically result in a loss of channel function, the S140G mutation resulted in a gain of channel function. In cultured cells, expression of the S140G mutant channel resulted in dramatically enhanced potassium channel currents and markedly altered potassium channel gating kinetics, changes that would be predicted to reduce the probability of fibrillatory conduction. These findings, and the abnormal prolongation of ventricular repolarization observed in these same individuals, suggest that there may be disparate effects of the gain of function mutations on atrial and ventricular physiology, possibly through differential interactions with distinct partner proteins or through other unknown mechanisms. In addition, both sympathetic and vagal stimulation may cause reductions in effective refractory period (ERP) (Takei et al., 1991) and thus influence the maintenance of AF. Acetycholine activates I*_KACH_*-channels via G-protein second messengers, resulting again in decreased ERP and action potential duration (APD) (Ohmoto-Sekine et al., 1999). It is postulated that norepinephrine lowers ERP by activating IP3 and diacyl glycerol leading to an initial influx of Ca^2+^. Finally, I*_KH_*, an inward K^+^-conductance current, has also shown to be increased under both sympathetic and parasympathetic stimulation (Ehrlich et al., 2004).

Cell coupling also may play a role in the propensity to AF. PVs exhibit a reduced density of connexin-40 (Cx-40) within their myocardial sleeves as compared with adjoining left atrial tissue (Verheule et al., 2002). The implied decreased electrical coupling of cardiocytes would result in lowered conduction velocity, promoting AF maintenance. Further support for a role of connexins in AF comes from mice lacking Cx-40 which have been shown to have both conduction block and increased AF development (van Rijen et al., 2001). The existing human data are, however, somewhat less convincing with only occasional reports of somatic mutation in the connexin genes and no definitive evidence of heritable defects in cell-cell coupling in AF (Gollob et al., 2006). While common genetic variation in the connexin genes may modulate electrical coupling between atrial and PV myocytes, a role for such variation in human AF remains unproven.

Primary structural differences in the PV and atrial cellular architecture may also sustain re-entry through effects on conduction velocity. Anisotropic conduction is highly dependent on the nature of the connections at specific cell boundaries (Sano et al., 1959). Conduction delays occur in the left superior PV and have been correlated with myocyte fiber orientation (Hocini et al., 2002; Hamabe et al., 2003). Similarly, discrete effects on myocardial architecture (structural and functional) may cause the disorganized propagation of an impulse to neighboring tissue, resulting in chaotic rather than focal, linear conduction (de Bakker et al., 2002). Connective tissue differences may also contribute to the primary electrophysiologic substrate in AF but are often among the most obvious differences in atria remodeled by chronic AF (Spach and Dolber, 1986; Hassink et al., 2003). Physiologic connective tissue barriers are seen in PVs and are associated with impulse conduction block or delay (Hamabe et al., 2003).

These structural differences may have some developmental underpinnings. For example, it has been noted that transgenic mice with increased levels of TGF-β 1 had increased baseline connective tissue within their atria and were consequently predisposed to AF development (Verheule et al., 2004). Numerous Mendelian cardiac and vascular diseases associated with AF are now known to result from inherited perturbations of TGF-β signaling and it is certainly conceivable that subtle variation in these same pathways during PV angiogenesis and atrial patterning may contribute to abnormal myocyte networks that sustain AF.

## Perpetuation of AF: atrial remodeling

“Atrial remodeling” is a concept which by the presence of AF affects the structure and function of the atria in a manner that favors the maintenance of the arrhythmia (Wijffels et al., 1995). How does the development of this remodeling perpetuate AF and perhaps more importantly is some element of the propensity to atrial remodeling a core component of the intrinsic predisposition toward AF? Studies have noted slowing of impulse conduction and reduced ERP in atrial cells after AF (Gaspo et al., 1997). Atrial tachycardia has been shown to reduce ERP primarily through a decrease in the I_Ca2+−L_–current of myocytes. In addition, sustained fibrillation has also been associated with structural changes such as myocyte hypertrophy, myocyte death, and atrial stretch (Aime-Sempe et al., 1999), which act in concert to reduce conduction velocity. Both electrophysiological and structural changes of the atria are thought to be involved in the development of chronic AF, and so the theory that “AF begets AF” has been introduced. If this concept is dissected further, then one notes that PVs are the primary source for ectopic foci during AF. Therefore, it is likely that the arrhythmogenic activity of PVs may be an important cause of “atrial remodeling.” Thomas et al. showed that the “Star” model for radio-frequency ablation, where PVs are electrically isolated, resulted in “reverse atrial remodeling” in patients with AF (Thomas et al., 2003). The study found that atrial size and function was restored after surgery. In addition, structural changes in the atria after remodeling, such as stretch, may also result in increased PV activity. Atrial stretch leads to increased intra-atrial pressure causing a rise in the rate and spatio-temporal organization of electrical waves originating in the PVs (Kalifa et al., 2003). Rapid atrial pacing (RAP) has also been shown to reduce ERP and APD within PV myocytes. RAP reduces the density of I_*Ca*2+−_*_L_*-and I*_To_*-currents, both involved in the plateau phase of action potentials (Chen et al., 2001). The same study found that PV myocytes had increased I*_f_*-currents after RAP. These changes imply that electrical and structural remodeling increase the likelihood of ectopic PV automaticity and AF maintenance and provide a potential mechanism by which atrial remodeling might take place.

The process of remodeling itself may be an intrinsic part of cardiac development. Early plasticity has numerous evolutionary advantages and is observed in many tissues (even exploiting pathways already implicated in cardiac arrhythmias) (Salinas and Price, 2005), though whether it operates in the heart remains an open question. These existing questions suggest that the exploration of developmental electrophysiology is likely to have a significant impact on our understanding of disease biology in adult arrhythmias and sudden death.

## Modulation of AF: disease states

Given the proposed role of developmental factors in AF, it is likely that many of the disease states associated with AF also act at this level. To date few studies have explored these relationships. For example, there is a strong reciprocal epidemiologic relationship between heart failure and AF that is unexplained (Wang et al., 2003). Some forms of heart failure are known to result from changes in the dynamics of calcium handling in cardiomyocytes and similar effects have been associated with increased automaticity in cells within the PVs (Honjo et al., 2003), but no direct mechanistic connections have yet been made between these two common syndromes. The syndrome of tachycardia-related cardiomyopathy that is seen in AF might be interpreted as a shared common diathesis to AF and heart failure, since the very presence of sustained AF suggests a primary abnormality of myocardium. Whether in some instances this reflects a shared aberrant remodeling response is not known, but as functional imaging tools improve these hypotheses can be directly tested.

Similarly, thyroid hormone signaling, can also be connected with acute effects on triggered activity in the myocardial sleeves of the PVs (Chen et al., 2002b), but the role of chronic subclinical thyroid imbalance during cardiogenesis not known. Inflammatory cells are critical for the tissue remodeling that takes place during development, but while there are clear effects on AF in later life, the role of these same pathways in patterning atrial biology is just beginning to emerge (Rudolph et al., 2010; Issac et al., 2007). Fundamental relationships between metabolic abnormalities, including obesity, and AF are also recognized. Indeed, metabolic abnormalities are emerging as some of the most rapid changes seen in atrial remodeling (Nattel, 2004). The intrinsic differences in metabolic response seen in AF, and the relationship with obesity, suggest that there may be both inherited and acquired components to this aspect of the AF diathesis ranging from distinctive regulation of core body temperature to biases in substrate utilization (Chen et al., 2003b). Finally, transgenerational differences in autonomic reactivity have been documented in several species. The role of early physiology in patterning these differences is emerging, and the extensive innervation of PVs by sympathetic and parasympathetic nerves makes a role for these effects in AF plausible, but there are no data that directly address this hypothesis (Gardner and O'Rahilly, 1976).

Finally, the link between AF and stroke is multifaceted with AF predicting an increased risk not only of thromboembolic stroke, but also of hemorrhagic stroke even in those who are not on any anticoagulant therapies. This connection has no clear mechanistic basis, but the increasingly recognized role of endocardial signals in cardiac development proposes at least one potential connection, a shared upstream endocardial abnormality.

## Conclusion

AF is a highly complex disease with numerous contributing factors. In this review, we have briefly summarized the data suggesting a significant role for developmental factors in the genesis of the underlying diathesis to this common arrhythmia. We anticipate that the study of the developmental basis of adult onset arrhythmic disorders will complement existing approaches to the biology, diagnosis, and therapy of these clinically important syndromes.

## Funding sources

NIH, Harvard Stem Cell Institute, Leducq Foundation

### Conflict of interest statement

The authors declare that the research was conducted in the absence of any commercial or financial relationships that could be construed as a potential conflict of interest.
